# Impact of Guidance and Multitasking on Manual Dexterity Skills in Dentistry

**DOI:** 10.1055/s-0042-1743155

**Published:** 2022-04-18

**Authors:** Mohamed El-Kishawi, Khaled Khalaf, Colin Murray, Ruba Odeh, Tracey Winning

**Affiliations:** 1 College of Dental Medicine, University of Sharjah, Sharjah, United Arab Emirates; 2 Institute of Dentistry, University of Aberdeen, Aberdeen, United Kingdom; 3 College of Dentistry, Ajman University, Ajman, United Arab Emirates; 4 School of Dentistry, The University of Adelaide, Adelaide, South Australia

**Keywords:** motor skills, manual dexterity, learning by guidance, simulation, dentistry

## Abstract

**Objectives** This study investigated the effect of learning by observation on the development of fine motor skills related to endodontic manual instrumentation. We evaluated if learning by observation with guidance had any influence upon operator performance under tense or taxing conditions.

**Materials and Methods** Dental students prepared standardized simulated root canals of varying morphology. Learning involved silent video with hand guidance ( *n* = 23), audiovisual combined with oral instructions ( *n* = 23), or silent video ( *n* = 13). Undergraduates who previously completed conventional preclinical endodontics provided comparative data as a control group ( *n* = 16). During investigations, a root canal of a lower molar plastic tooth was shaped, beginning with a primary task, and followed by multitasking conditions. The performance of the students was assessed by evaluating the accuracy of dental canal shaping and time taken to complete the task.

**Statistical Analysis** Differences were analyzed using ANOVA ( *p* < 0.05).

**Results** Performance was similar during learning between the three experimental groups. Accuracy of the performance did not differ within each group for the two tests nor between the groups at each test ( *p* > 0.05).

**Conclusions** These findings demonstrated that performance subsequent to learning by observation without instructions was comparable to learning with instructed observation. The results also identified that the performance of the experimental group (1.5- to 2-hour practice) was comparable with the conventional control group (15- to 20-hour practice). Alternative approaches to learning dexterity skills in dentistry may provide improved outcomes, especially in demanding situations.

## Introduction

Observational practice is considered to be an effective method for learning simple and fine motor skills. 1 2 It is defined as learning by observing an individual person undertaking key spatial and/or temporal components of a task and subsequently producing a cognitive representation of the action pattern. 3 Observation, in the context of demonstration by an expert, is a common tool for supporting procedural knowledge development for simulated and clinical activities in dentistry. 4 5

Observational practice may not be perceived as effective as physical practice. Nevertheless, it has a significant impact upon the processes of learning, especially when combined with physical practice. 6 7 This impact has been observed at both neurological and behavioral levels. 8 It is suggested that processing mechanisms associated with physical movement and observation are mediated via similar processes. 9 Observational practice can help the learner to extract vital information related to requirements and coordination of a motor task, which offers the learner the opportunity to process and evaluate the task cognitively prior to physical performance of the procedure. This additional processing can be represented when learners take turns in physical and observational practice in pairs. Such an example may be student/assistant situation in four-handed dentistry. 9 This form of practice has been determined to contribute significantly to the motor skill learning processes in addition to being relatively cost-effective. 10

Demonstration is a very common method of providing information about how to perform a motor task. However, there has been limited research on the most appropriate way to utilize demonstrations as an effective instructional strategy for motor skill learning. 11 12 Nevertheless, there has been increasing interest by researchers in using observational learning as an implicit learning approach. 1 13

Implicit approaches, which require minimal conscious involvement, reduce the effect of self-focus and self-regulation on learning and subsequent performance. 14 Such an approach may lead to positive and consistent outcomes, even with stressful conditions when compared with frequently employed explicit methods of learning and teaching. 15 Outcomes associated with implicit learning are of importance in clinical dentistry because working under stressful circumstances, whether it be psychological or physiological, is a common characteristic during either undergraduate studies or employment in a dental setting. 16 17 These stressors may be of significance as they can compromise the clinical performance of undergraduate dental students 18 and may negatively impact on their learning experiences. 19 It has been proposed that learning implicitly in a preclinical environment can minimize loss of performance prior to involvement in the clinic. 1

Another implicit approach for learning involves learning from observation. For example, observation combined with guidance, known as guided observation (GO), involves learning motor skills via a nonverbal method with limited conscious awareness of what is learnt and how a motor task is executed. 3 Consequently, it is difficult for the learner to provide verbal details on how a motor task is undertaken and therefore is considered to be an implicit way of learning. 20 GO reduces performance errors by using physical guidance to the direct movements, thereby reducing the need for conscious correction of mistakes by the performer. Conversely, learning from observation in combination with instructions, such as instructed observation (IO), involves the acquisition of motor skills with supplementary verbal instructions and high conscious awareness by the performer on how a motor task is executed. Thereby, the learner can provide detailed verbal steps about a motor task, having learnt explicitly. 20 Studies identify that GO encourages more stable performance under stressful conditions and when multitasking 20 21 as opposed to learning by IO.

Visual demonstrations of simulated endodontic techniques and procedures during teaching have been supported by the Australian Society of Endodontology (ASE). 22 Therefore, further studies to investigate effective methods for learning and teaching dental fine motor skills are required, using conditions that produce robust performance, even under demanding situations. 23

This study intended to investigate the influence of observational learning delivered by GO, IO, or observation only (OO) on the development of fine motor skills related to root canal hand instrumentation. 1 It was hypothesized that learners who used a GO approach would experience minimal deterioration in performance under multitasking conditions. In contrast, it was anticipated that learners who completed IO and OO tasks would demonstrate a reduction in performance under multitasking conditions.

## Materials and Methods

### Study Groups

**Fig. 1:**
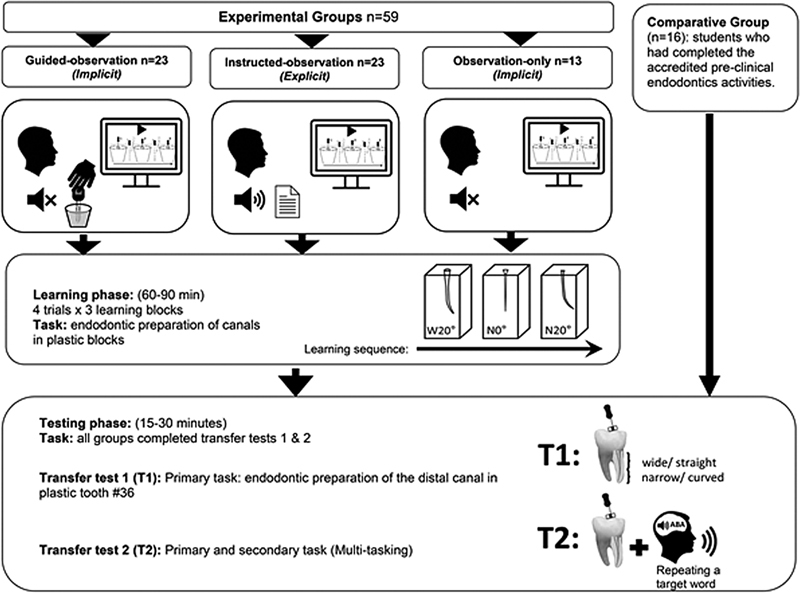
Summary of the study plan. W20°, wide/curved; N0°, narrow/straight; N20°, narrow/curved (S4-U1–20°, S8-BS2-U, and S8-BC2-U, Nissin Dental, Minami-ku, Kyoto, Japan).

The last two years of the BDS program at the University of Adelaide were invited to take part in this study ( *n* = 76; Ethical Approval no. H-2012- 117). The main investigator explained the study to the participants during a scheduled meeting. Subsequently, informed consent was obtained from all volunteers. Consequently, 59 third-year undergraduates were assigned at random to three groups ( Fig. 1 , Table 1 ). Study participants in the experimental learning groups had no previous endodontic teaching to bias study results. 15 Manual skills at baseline was not assessed, to prevent accumulation of verbalizable technical knowledge by the study volunteers. 24 Performance levels of the GO, IO, or OO learning groups were compared with 4th year of the BDS Program ( Fig. 1 , Table 1 ). These fourth-year students had previously completed conventional preclinical endodontic learning and teaching. This facilitated comparison of performance for those in the observational test cohorts against student performance for those who had finished a conventional skills course in endodontics, the control group.

**Table 1:** Summary of demographic data for each group

	GO group		IO group		OO group		COM group	
	( *n* = 23)		( *n* = 23)		( *n* = 13)		( *n* = 16)	
	*n*	%	*n*	%	*n*	%	*n*	%
Gender								
Male	15	65.2	10	43.5	5	38.5	5	31.3
Female	8	34.8	13	56.5	8	61.5	11	68.8
Handedness								
Right	23	100	21	91.3	12	92.3	14	87.5
Left	0	00.0	2	08.7	1	07.7	2	12.5
Vision								
Normal	12	52.2	10	43.5	4	30.8	10	62.5
Corrected Glasses	6	26.1	7	30.4	7	53.8	2	12.5
Corrected Contacts	5	21.7	6	26.1	2	15.4	4	25.0
Age group (y)								
20–24	16	69.6	20	87.0	12	92.3	14	87.5
> 24	7	30.4	3	13.0	1	07.7	2	12.5

Abbreviations: COM, comparative; GO, guided observation; IO, instructed observation; OO, observation only.

### Materials for the Two Stages of the Study

A pilot study of novice students in endodontics ( *n* = 8) was undertaken to establish (1) the time to be set for each exercise, (2) key directions for the use of endodontic hand files to minimize provision of procedural knowledge, (3) type and sequence of endodontic learning blocks, (4) preference of appropriate endodontic manual files for experimental groups, and (5) how many tests would be required to demonstrate increasing skill. From this pilot study, three endodontic blocks were chosen for the first phase ( Fig. 1 ) and enclosed within silicon material. Thereby, study volunteers were unaware of the simulated morphology of the root canal and the way to move the file, aiming to minimize hypothesis testing. The chosen blocks were broad/curved 20 degrees; narrow/unbent; and narrow/curved 20 degrees ( Fig. 1 ; S4-U1–20°, S8-BS2-U, S8-BC2-U, Nissin Dental, Minami-ku, Kyoto, Japan). The blocks were sequenced to minimize the introduction of mistakes by starting with the most straightforward exercise and advancing toward the most challenging procedure. 15 The information given to study participants was limited to minimize loading on the working memory. 25

In the first stage, exercises 1 and 2 required preparation of the distal root canal of an artificial lower left first molar tooth (B22X-END#36, Nissin Dental, Minami-ku, Kyoto, Japan), which mimics the morphology of the natural tooth, with the distal canal being wide at the entrance and then curving (∼20 degrees) before narrowing toward the root apex. Therefore, the task was considered to be of medium complexity. Two artificial teeth were placed in customized jaw bases used in a bench-mounted phantom head (Frasaco, United States).

During testing and learning stages, study members prepared dental root canals with Nickel titanium (NiTi) root canal manual files. Working length was established and maintained for all manual files used during the initial (17.5 mm) and final (19.5 mm) phases. The same manual instruments were used in both phases. Root canal length was confirmed with the aid of digital radiographs and a digital imaging system and the radiographs were analyzed using digital radiographic imaging software (Exact V10, Software of Excellence, Australia). The curvatures of simulated root canal were recorded with radiographic images using an imageJ software package (ImageJ v1.47, National Institutes of Health, United States). 26

### Learning (Phase 1) and Testing (Phase 2) Procedures

Phase 1 was based upon pilot study data and participants were requested to prepare standardized canals by a crown-down procedure using artificial blocks for four times of each of three blocks ( Fig. 1 ). The average time taken to complete the preparation of the simulated canals was 60 minutes.

### Video Demonstration

**Fig. 2:**
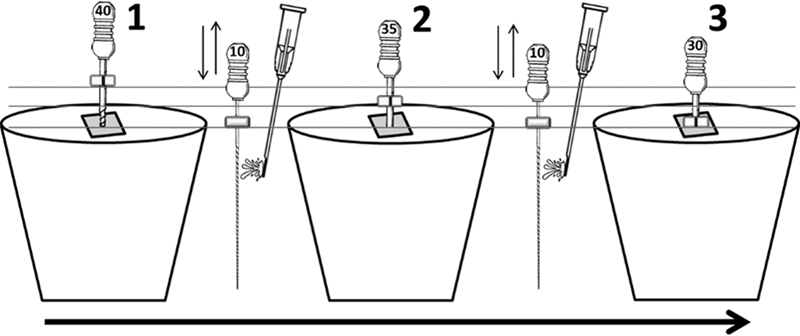
Diagram used in the second section of the video to indicate the progress of each instrument. *Horizontal lines* are shown on this diagram to indicate the difference in the depth of the hand instruments but were not shown in the video.

Key movements and images demonstrating the preparation steps were presented by video. Video demonstrations were edited using Windows Movie Maker software (Windows movie maker, V16.4, 2012, Microsoft Corporation, United States). The video contained three sections. The first section was a video of all the steps in the balanced force technique using one hand instrument. This was repeated three times to show advancement of the instrument down the simulated dental root canal. The second section was a step-by-step demonstration, supported by images ( Fig. 2 ) of the key movements and demonstrated cleansing of the canal. The third section demonstrated the balanced force technique on one plastic block using the last hand instrument used in preparing the full length of the canal. This section demonstrated the rubber-stopper touching the top surface of the block and that the instrument was free inside the canal. This was represented by three free insertions and removals of the hand instrument from the canal.

When participants in the GO group watched the video, the researcher placed his hand over the participants' fingers to imitate the three key movements of the balanced force technique. This was achieved using a large metal screw fitted with an acrylic handle (hand instrument) placed in a plastic cup filled with periphery wax (Surgident, United States).

The IO group watched the same video as the GO group supplemented with audio/verbal instructions. Verbal audio instructions consisted of three sections. The first contained instructions about the balanced force technique. The second showed a diagram ( Fig. 2 ) indicating the progress of the three hand instruments down the canal. Instructions in this section consisted of a step-by-step description of the basic balanced force technique and the cleaning process between each hand instrument. The final section of instructions included a repeat of the balanced force technique instructions including cleaning and ending with a description of the position and adaptation of the final instrument inside the root canal. The OO group watched the same video as the GO group, but without any physical guidance or audio or written instructions.

### Transfer Test 1: Primary Task Condition

Study volunteers were advised they needed to complete this exercise and shape the simulated root canal, as undertaken in the learning phase. Participants were then provided with digital radiographic images of both the tooth and distal root canal. Finishing time was noted from when the first instrument was inserted into the canal until completion of the task or until reaching the maximum 12 minutes time limit.

### Transfer Test 2: Primary and Secondary Task Conditions

Study volunteers were asked to perform the same primary task as in transfer test 1 within a limited time as best as they could while they completed a secondary task. The secondary task entailed listening to an audio recording randomly playing six dentally related expressions comprising open, wider, light, irrigation, mirror, and suction, at intervals of 2 seconds. Study volunteers were asked to remember and reiterate the word that was mentioned prior to the key word “suction.” 27

### Performance Assessment

#### Quality of Root Canal Preparation

The quality of root canal preparation was examined by assessing the last instrument in the prepared canal in relation to working length and its canal adaptation as previously reported by El-Kishawi et al. 15 Learning phase blocks with prepared root canals and inserted instruments were digitally radiographed. Canal preparations were evaluated by ImageJ software through calculating the difference between the prepared canal and the working length (17.5 mm). The accuracy of canal preparations of participant learning blocks was assessed as the mean distance in millimeters (mm) that the final instrument reached within the root canal in comparison to canal working length.

The quality of canal preparations in transfer tests 1 and 2 was determined by recording the distance in millimeters (mm) that the final instrument reached in comparison to the distal canal working length (19.5 mm; Fig. 2 ). Tooth position on the digital scale and during radiographic exposure was standardized by using two vinyl polysiloxane putty keys (Laboratory-Putty, Coltene-Whaledent, United States).

#### Completion Time and Procedural Errors

The time required to finish canal preparations for each experimental group was recorded in minutes. Procedural errors including canal blockages, ledges, or file fracture were assessed with a dental operating microscope and by using digital radiography. The number of sequence rules related to the preparation method mentioned by study volunteers following the exercise was recorded for every study volunteer. Mean values and standard deviations were obtained for the experimental observational groups. Performance levels of the three learning groups were then compared with the fourth-year comparative (COM) group (see Table 1 ).

Tests of intra- and inter-rater reliability were assessed by double determination on a random sample of 5 to 20% of the experiments involving the first author and an endodontic specialist who assessed at random 51 artificial blocks and 27 teeth. The quality of root canal shaping was recorded in addition to the number and type of procedural errors such as canal blockage, ledge formation, and file separation.

### Statistical Analysis

Significant differences in the outcome measures were tested using analysis of variance (ANOVA), the Kruskal–Wallis test ( *H* ), and chi-squared ( *χ* ^2^ ) test depending on the outcome measure and the type of data analyzed. All data analyses were undertaken using SPSS (version 20.0; SPSS Inc, Chicago, Illinois, United States), and statistical significance for quantitative and categorical data was set at *p* < 0.05. Effect size (Cohen's *d* ) were calculated using the method described by Durlak. 28

## Results

Data from 75 study volunteers (35 males and 40 females) were analyzed. Tests of intra- and inter-rater reliability demonstrated no statistical differences between measurements ( *p* > 0.05) and a high level of intra- and inter-rater agreement (Intraclass Correlation Coefficient [ICC] > 0.8).

**Fig. 3:**
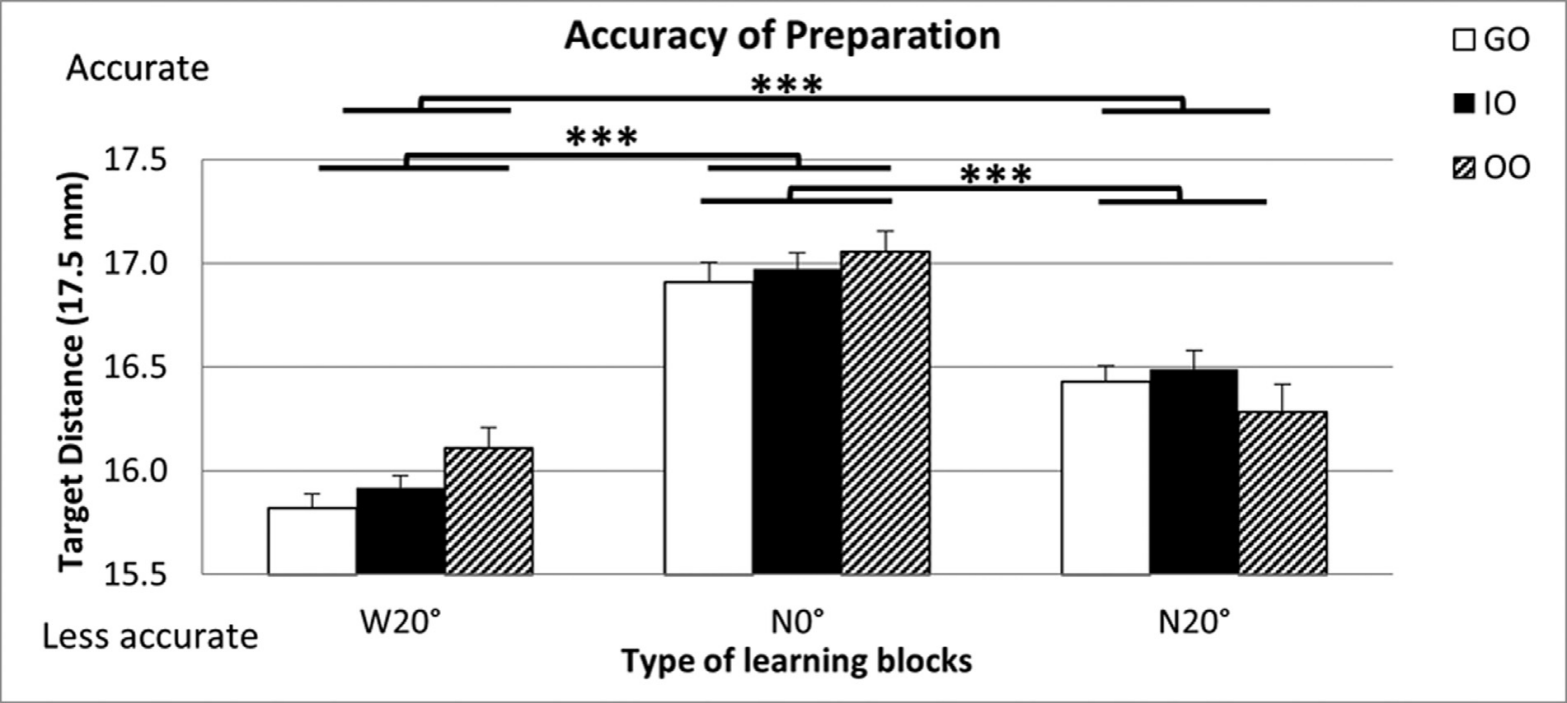
Mean distance (millimeters) that the last instrument reached compared with the target distance (17.5 mm) for the prepared canal in each of the endodontic learning blocks for the GO, IO, and OO groups (GO, guided observation; IO, instructed observation; OO, observation only). Decrease in length = reduced accuracy; error bars = +1 standard error mean. W20° = wide/20-degree curved canal; N0° = narrow/straight canal; N20° = narrow/20-degree curved canal. Sequence of learning trials for all groups was from block W20° to block N20°. ***Significant difference between learning blocks, *p* < 0.001.

For the GO group, the mean preparation lengths in the three learning blocks were from 15.76 to 17.41 mm ( *M* = 16.72 mm, SD = 0.38). For the IO group, the mean prepared length for the learning blocks ranged between 15.64 and 17.52 mm ( *M* = 16.80 mm, SD = 0.37). For the OO group, the mean length of the learning blocks ranged between 15.68 and 17.72 mm ( *M* = 16.82 mm, SD = 0.40; Fig. 3 ). A group X learning block (3 × 3) repeated measure ANOVA revealed no significant differences in accuracy of canal preparation between the three experimental groups, *F* (2, 56) = 0.74, *p* = 0.484. Differences in accuracy of preparation within learning blocks across all three experimental cohorts were statistically significant, *F* (1, 56) = 41.76, *p* < 0.001. Furthermore, it was found a significantly higher accuracy of preparation for the narrow/straight (N0°) block compared with the wide/curved (W20°; *p* < 0.001, *d* = 2.87), and the narrow/curved (N20°; *p* < 0.001, *d* = –1.35) blocks. Results also showed a significantly higher accuracy of preparation for the N20° block compared with the W20° block ( *p* < 0.001, *d* = 1.33).

**Fig. 4:**
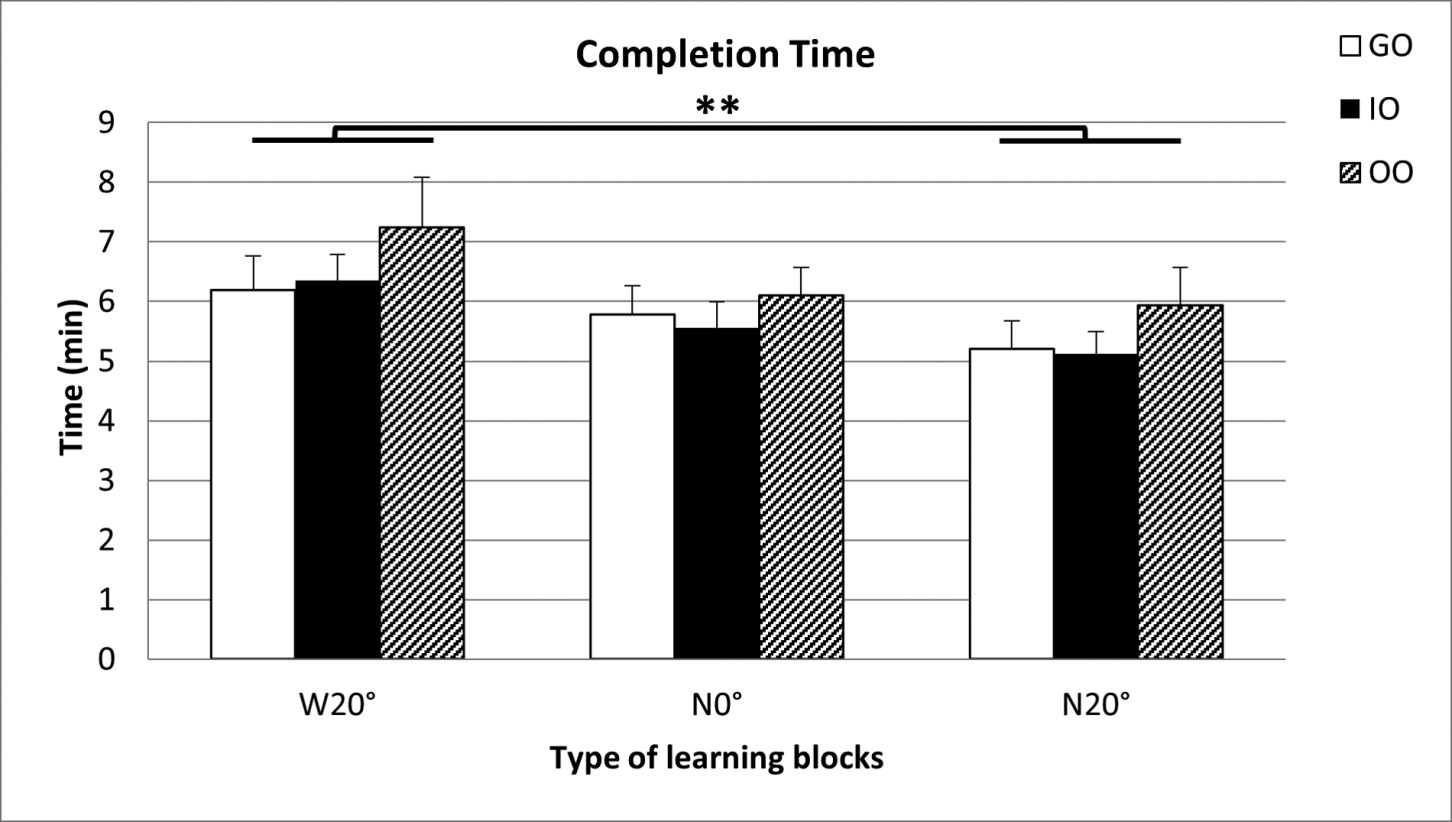
Mean time (minutes) taken to complete canal preparations for each type of the endodontic learning blocks for the GO, IO, and OO groups (GO, guided observation; IO, instructed observation; OO, observation only; W20°, wide/20-degree curved canal; N0°, narrow/straight canal; N20°, narrow/20-degree curved canal). Sequence of learning trials for all groups was from block W20° to block N20°; error bars = +1 standard error mean. **Significant difference between learning blocks W20° and N20° ( *p* < 0.01).

For the GO group, the mean preparation times for each of the three learning blocks ranged from 1.27 to 12.78 minutes ( *M* = 5.72 minutes, SD = 2.45); for the IO group, they ranged between 1.90 and 10.73 minutes ( *M* = 5.68 minutes, SD = 1.98); and for the OO group, they ranged between 2.63 and 12.14 minutes ( *M* = 6.42 minutes, SD = 2.33; Fig. 4 ). A group X learning block (3 × 3) repeated measure ANOVA revealed no significant difference in preparation times between the three experimental groups, *F* (2, 56) = 0.17, *p* = 0.95. Completion times between learning blocks across all the groups was significant, *F* (1, 56) = 5.01, *p* = 0.010 ( Fig. 4 ). A Bonferroni post hoc test revealed that participants spent significantly shorter time preparing the N20° blocks compared with the W20° block ( *p* < 0.001, *d* = 0.50).

**Fig. 5:**
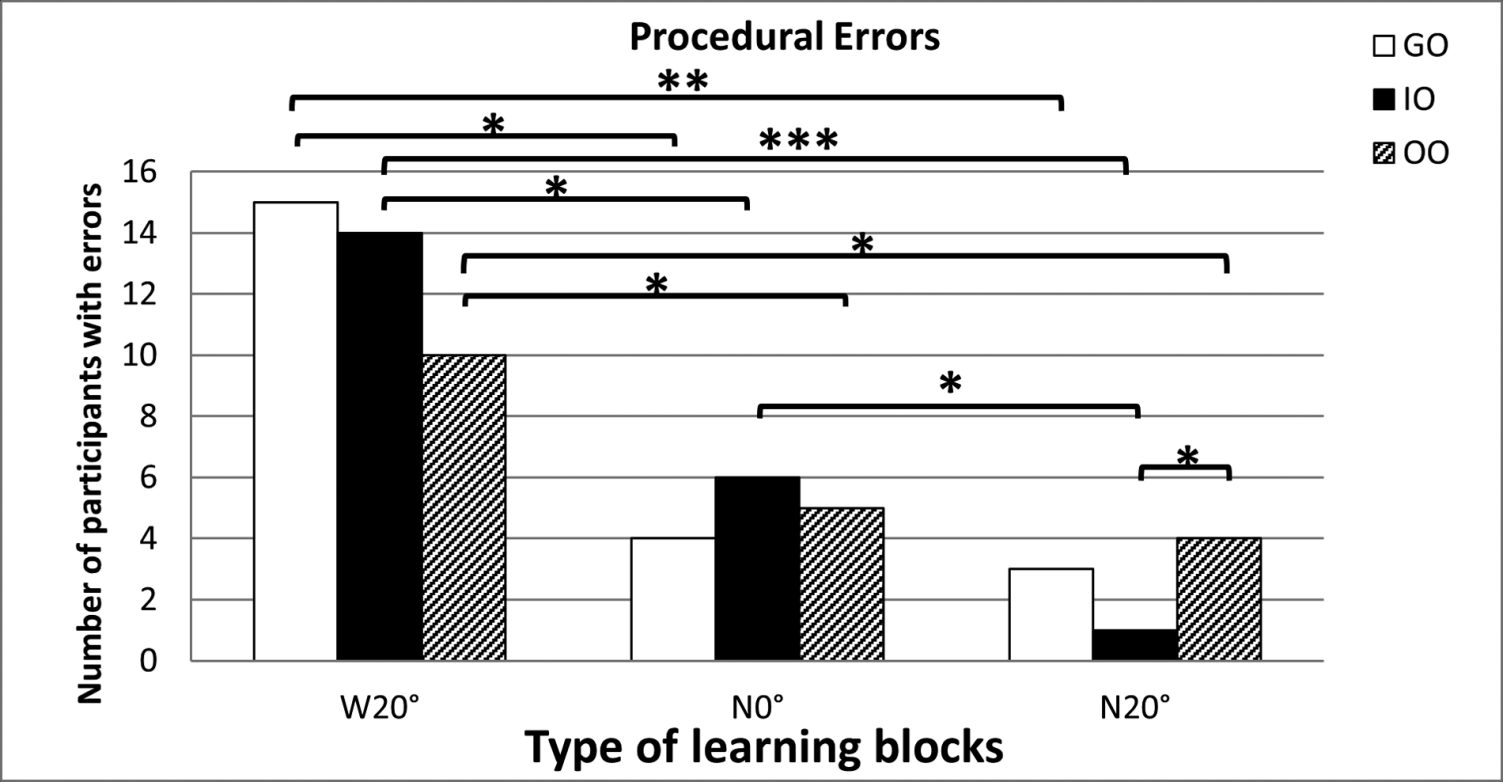
Number of procedural errors made by the GO, IO, and OO groups during the learning phase (GO, guided observation; IO, instructed observation; OO, observation only; W20°, wide/20-degree curved canal; N0° = narrow/straight canal; N20°, narrow/20-degree curved canal). Significant difference between learning blocks: * *p* < 0.05; ** *p* < 0.01; *** *p* < 0.001.

During the learning trials, procedural errors ranged between root canal blockages ( *n* = 47, 68% of errors), ledges ( *n* = 2, 3% of errors), canal transfer ( *n* = 2, 3% of errors), file fracture ( *n* = 16, 23% of errors), or fractured roots ( *n* = 2, 3% of errors). The total number of errors made by experimental groups was 69 errors from 62 study volunteers (10% of the learning blocks; Fig. 5 ). The Kruskal–Wallis test ( *H* ) showed no significant differences in the number of procedural errors among the three groups ( *H* (2) = 1.69, *p* = 0.51). *Z* -test results showed that the IO group had a significantly fewer procedural errors in block N20° compared with the OO group (95% confidence interval [CI]: –0.03 to –0.50; *z* = –2.20; *p* = 0.03). Significant differences between blocks within each group are presented in Table 2 .

**Table 2:** Comparisons of procedural errors between learning blocks within each experimental group

Group	Comparison bock	Reference block	*n*	*Z* -value	*Z* -test *p* value	95% confidence intervals	
GO	W20	N0	23	3.29	0.001	0.76	0.19
GO	W20	N20	23	3.63	< 0.001	0.81	0.24
IO	W20	N0	23	2.38	0.02	0.63	0.06
IO	W20	N20	23	4.09	< 0.001	0.84	0.29
IO	N0	N20	23	2.05	0.04	0.43	0.01
OO	W20	N0	13	1.99	0.047	0.76	0.01
OO	W20	N20	13	2.36	0.02	0.85	0.08

Abbreviations: GO, guided observation; IO, instructed observation; N0°, narrow/straight canal; N20°, narrow/20-degree curved canal; OO, observation only; W20°, wide/20-degree curved canal.

The mean preparation length in transfer tests for the GO, IO, OO, and Comparative (COM) groups were from 15.21 to 18.93 mm ( *M* = 17.03 mm, SD = 0.89), 15.61 and 18.76 mm ( *M* = 17.05 mm, SD = 0.85), 15.66 and 19.11 mm ( *M* = 17.06 mm, SD = 0.83), and 14.67 and 18.97 mm ( *M* = 16.97 mm, SD = 0.97), respectively. A group X transfer test (4 × 2) repeated measure ANOVA revealed no significant differences in quality of preparation between the two transfer tests for each group, *F* (1, 71) = 1.37, *p* = 0.25. Results also showed no significant difference in quality of preparation between the four groups at each transfer test, *F* (3, 71) = 0.11, *p* = 0.96.

**Fig. 6:**
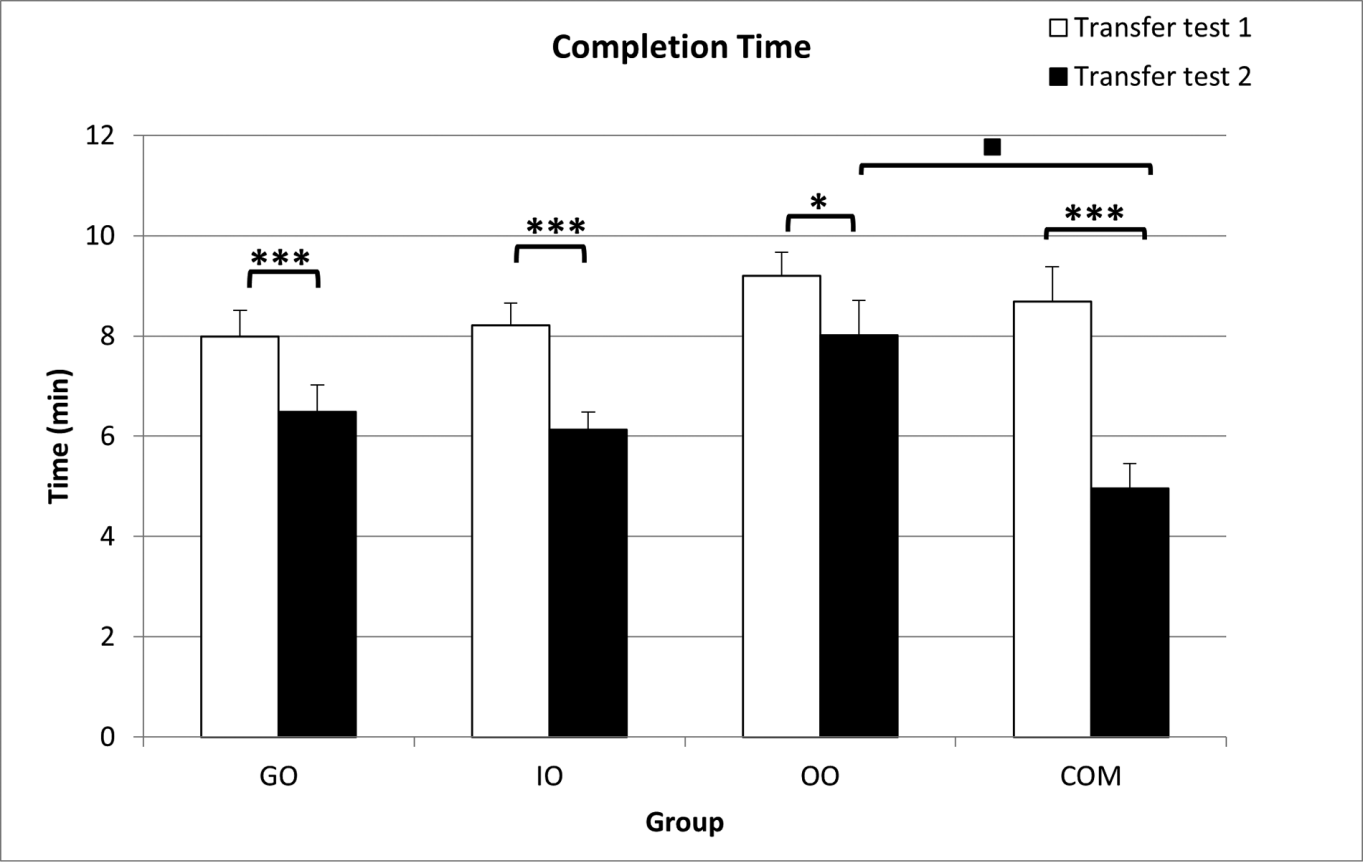
Mean time (minutes) spent to complete the task during transfer tests 1 and 2 for the GO, IO, OO, and COM groups (GO, guided observation; IO, instructed observation; OO, observation only; COM, comparative; error bars = +1 standard error mean). ***Significant difference between transfer tests at each group ( *p* < 0.001). ▪Significant difference in transfer tests 2 between the OO and COM groups ( *p* < 0.05).

For the GO, IO, OO, and COM groups, the mean completion time during the two transfer tests ranged between 1.73 and 12.00 minutes ( *M* = 6.38 minutes, SD = 2.36), 2.05 and 12.00 minutes ( *M* = 6.26 minutes, SD = 1.75), 1.92 and 13.38 minutes ( *M* = 7.66 minutes, SD = 1.96), and 1.42 and 12.30 minutes ( *M* = 5.75 minutes, SD = 2.01), respectively ( Fig. 6 ). A group X transfer test (4 × 2) repeated measure ANOVA revealed a significant difference in completion time between each of the two transfer tests across the four groups, *F* (1, 71) = 342.16, *p* < 0.001. A Bonferroni post hoc test revealed significant differences between transfer tests 1 and 2 ( Fig. 6 ).

Completion times between the four groups revealed a significant difference, *F* (3, 71) = 2.69, *p* = 0.049. A Games–Howell post hoc test showed that the COM group took significantly less time to complete canal preparation than the OO group at transfer test 2, *F* (3, 71) = 4.85, *p* = 0.004, *d* = 1.44 ( Fig. 6 ).

All groups demonstrated a high percentage of correct responses during the secondary task condition; for the GO group, it was 90% (SD = 0.11) compared with 94% (SD = 0.06) for the IO group, 93% (SD = 0.07) for the OO group, and 95% (SD = 0.08) for the COM cohort. Chi-squared ( *χ* ^2^ ) test results showed no significant differences between groups, *χ* ^2^ (63) = 66.60, *N* = 75, *p* = 0.35.

During the transfer tests, procedural errors involved root canal blockages ( *n* = 15, 54% of errors), ledge development ( *n* = 8, 28% of errors), and root fractures ( *n* = 5, 18% of errors). A total of 28 errors were made by 28 participants within the three experimental and COM groups (12% of the transfer teeth). While there were variations in the number of errors produced by the different groups, the Kruskal–Wallis test (H) established these were not significantly different ( *H* (2) = 1.22, *p* = 0.59), nor were there significant differences in the number of procedural errors made by the three groups ( *H* (3) = 3.80, *p* = 0.31). The *Z* -test results showed there were no significant differences between the three transfer tests nor within or between groups for each test ( *p* > 0.05).

For the GO, IO, and OO groups, the average number of rules related to the balance force method ranged between 0 and 9 rules ( *M* = 5.91, SD = 2.52), 4 and 11 rules ( *M* = 8.96, SD = 1.80), and 0 and 9 rules ( *M* = 3.85, SD = 2.85), respectively. A one way ANOVA of the number of reported rules in the three experimental groups showed a significant effect between groups ( *F* (2, 58) = 21.52, *p* < 0.001). A Games–Howell post hoc test demonstrated a significantly higher number of reported rules by the IO group compared with the GO group ( *p* < 0.001, *d* = 1.42), and by the IO group in comparison to the OO group ( *p* < 0.001, *d* = 2.36). However, differences between the GO and OO groups were not statistically significant ( *p* = 0.10).

## Discussion

This study evaluated the influence of learning by observation on the development of fine motor skills used during hand instrumentation of simulated dental root canals. Observational learning utilized in the current study involved GO, IO, or OO. Observation, in the context of demonstration by an expert, is a common tool for supporting procedural knowledge development for simulated and clinical activities in dentistry and medicine. 5 29

During learning, quality of canal preparation and completion times in the three experimental groups (GO, IO, and OO) were similar over the course of preparing the learning blocks. While the IO cohort made significantly fewer procedural errors in blocks with a narrow/curved canal than the OO group, overall, all observation groups demonstrated improvement throughout the learning trials. Based on the similar level of performance achieved between GI, IO, and OO groups by the end of this experimental phase, it is suggested that study volunteers working under each condition were equally competent in performance and they had learnt key endodontic canal preparation skills during the learning trials. These findings are similar to those of Masters et al 20 who evaluated the effect of similar observational learning strategies on a surgical task (i.e., suturing and knot tying) and found a significant improvement in performance, measuring completion times and hand movements, for each group over the learning trials and no statistical differences between groups.

Given the variations in the complexity of the various endodontic blocks, there were significant differences in the quality of canal preparation and finishing times between the learning blocks within each group. Specifically, preparations of the curved canals, both wide and narrow, were less accurate than the narrow/straight canal, with the wide/curved canal having the poorest preparation. Finishing times fluctuated, with a reduction in time as participants learning trials progressed through the blocks.

These differences in quality and completion times between the blocks for all groups confirm that the level of difficulty across the blocks varied. Completion times for the second (narrow/straight) and third (narrow/curved) canals were similar. However, fewer procedural errors were evident with the narrow/curved canals in comparison to the wide/curved and narrow/straight canals. This might be related to a learning effect, resulting from practicing on each previous block with wide/curved or narrow/straight canals.

The large number of study volunteers displaying procedural errors when shaping the wide/curved canal may have also been associated with the bigger size of manual instruments used for the wide canal compared with the narrow canals. Participants in all groups made errors in the wide/curved canal block, suggesting that this first block performance may have shifted to explicit conscious processes (i.e., hypothesis testing). Accuracy of performance improved after this block, supported by the decrease in number of errors across all three groups. The balanced force technique is a complex technique and this may have contributed to the number of procedural errors produced by participants. However, as this was the technique used in the endodontic program, this technique was used to enable outcome comparison between groups.

During the testing phase, two transfer tests were used for the experimental and COM groups. Transfer test 1 involved root canal shaping of the distal canal of a mandibular first molar tooth, this being the primary task. Transfer test 2 involved the same primary task as in test 1, but, in addition, study volunteers were asked to recall a “target” word from a random series of words. Thereby, in transfer test 2, the study participants were multitasking.

In summary, all experimental groups achieved a similar level of performance after the learning phase as the COM group in terms of preparation accuracy, completion times, and procedural errors in transfer test 1. In the subsequent multitasking transfer test 2, performance related to accuracy of root canal shaping was maintained across all groups and differences in procedural errors were not evident. However, completion times within each observational learning group and COM group decreased significantly under multitasking conditions. The only significant differences between groups were longer completion times for the OO group than those for the COM group under multitasking condition. The IO group provided significantly more “root canal preparations rules” than either the GO or the OO group.

It was hypothesized that learning by observation, combined with physical guidance (GO), may result in stable performance under multitasking conditions. 20 In contrast, observing videos with instructions (IO) or without instructions (OO) was expected to promote hypothesis testing using working memory resources, which would lead to performance breakdown under multitasking conditions. 1 However, the level of accuracy between the observational learning groups was similar. This suggests that the GO group did not achieve an implicit learning paradigm as expected. Furthermore, the results suggest that all three experimental groups learnt more explicitly. This conclusion is supported by a lower-than-expected accuracy of preparation across the three experimental groups. These findings are not consistent with previous studies. 20 For example, Masters et al 20 found that performance of an IO group showed deterioration in performance (i.e., more hand movements and slower completion times) under multitasking conditions. However, this inconsistency might be explained by the simple nature of the task, suturing and knot-tying, being investigated in Masters et al's 20 study compared with the more complex root canal preparation task used in this study.

To explain these results, it is important to review how procedural information related to the root canal hand instrumentation task was presented to each experimental observation group and the impact of these presentations on working memory. The poor performance of the observational groups might be explained by the relatively high complexity of the components presented in the video. This included three segments with visual cues, which may have resulted in overloading working memory and subsequent performance breakdown. 1

Physical guidance used in this study involved both visuospatial perception and attention, and somatosensory information involving the receptors in muscles, tendons, and fingertips. 23 30 It is possible that the GO group may have paid less attention to the sensory feedback related to the task and instead focused on monitoring the video on the computer screen. 31 This increase in complexity may have led to loading working memory and compromising the learning effect, resulting in lower levels of performance.

Retention of performance of the IO group under multitasking conditions may be explained by the audio/visual video presentation of the hand instrumentation technique. Mousavi et al 32 suggested that the partially independent nature of visual and auditory working memory might be useful when multiple sources are required for understanding. Therefore, effective working memory can be increased by presenting material in a mixed format instead of a single format. For example, visual forms of presentation such as a written phrase and a picture alone are more likely to overload the visual processor. However, if the written material is given by speech, parts of the cognitive load can be moved to the auditory processor.

The decrease in completion times within each group in transfer test 2 could be related to learning or practice effects. 33 Another explanation is that study volunteers were requested to finish the task quicker than the previous task. It is possible that in this situation study subjects positively perceived the stress associated with working quicker. Results have demonstrated that study subjects develop adaptive physiological and cognitive responses to stress, 34 which can enable them to keep their performance level and therefore enhance their finishing times. The higher performance in terms of faster completion times by the COM group compared with the OO group under multitasking conditions may be related to their experience and a longer period of simulated practice.

The implementation of the secondary task for multitasking was effective in this study. This is supported by the very high percentage of correct words correctly provided during multitasking, suggesting that participants in both experimental and COM groups were using their working memory resources. However, access to fewer rules by the GO and OO groups compared with the IO group indicates that their level of hypothesis testing during root canal preparation tasks was limited. This suggests that the GO and OO forms of learning encouraged some degree of implicit motor learning compared with learning with instructions. In assessing a surgical suturing task, Masters et al 20 obtained similar results and noted that the GO and OO groups reported fewer movement-related knowledge compared with an IO group.

Despite the increased interest in the role of observational learning in motor skill acquisition and its use in dentistry, 4 35 there is limited research about possible strategies to optimize learning by modelling, or about the factors that influence its complexity and attentional demands. 30 For example, there is little information about the most appropriate timing and source of procedural information (i.e., verbal, audio, or visual) to be provided during motor learning, or about the frequency of demonstrations in the learning process.

In a recent review of experts' opinions on strategies that promote implicit and explicit motor learning, there was no consensus by the experts regarding the classification of observational learning strategies. 3

## Study Limitation

This study did not include a delayed retention test due to the limited time available for data collection and the timing of commencement and completion of the simulation course in endodontics during the third year of the undergraduate dentistry program. Other limitations include the nature of the motor task in this study and the participants involved.

### Implications for Practice

Learning via observation, in the context of demonstration by an expert, is a common choice for learning fine motor skills for simulated and clinical activities in dentistry. 4 5 Furthermore, visual illustrations of simulated root canal procedures and methods during learning have been promoted by the ASE guidelines for dental education in endodontics. 22 However, it is proposed that, in future, educators should concentrate upon the design of activities that aim to produce an implicit approach to learning, especially within the early stages of learning. It is recommended that educators should aim to simplify the presentation of the different components of a task to reduce the load on working memory. 1 30 It is also recommended to take advantage of the partially independent nature of visual and auditory working memory by presenting simplified video demonstrations with basic audio instructions (cues) about the motor skill task. Furthermore, emphasis should be placed on the importance of providing minimal instructions related to root canal procedures. These instructions should focus on the outcome of the movement rather than the movement itself. 9 Both the amount and the timing of instructions are likely to substantially influence the learning process.

Future studies need to test a range of observational methods that include different frequencies, timings, and sources of procedural knowledge, such as a mixture of audio and video instructions to achieve implicit learning. Further evaluation of the effect of stress upon transfer of simulation skills to the dental practice would be beneficial. Testing a range of root canal preparation methods, including simpler techniques, is suggested as it may allow students to learn essential instrumentation methods before advancing onto the more complex ones. Observational learning has been a commonly used approach in medical and dental education, but there is little research to back up its use. 36 Further controlled studies of observational learning for dental undergraduates are necessary, including testing alternative implicit learning paradigms when learning skills in endodontics. Such studies should improve outcomes, specifically in multitasking and demanding conditions.

## Conclusion

Accuracy of root canal formation and finishing times of the GO, IO, and OO groups were similar during learning. Furthermore, the three observational groups who had 1.5 to 2 hours of practice attained levels of performance that were similar to those demonstrated by students in the BDS endodontic program who had 15 to 20 hours of practice. When tested, accuracy of canal preparation did not differ significantly within each of the observation and COM groups across the primary task (transfer test 1) and multitasking (transfer test 2) conditions, nor between the groups at each transfer test. This suggests that learning through physical guidance did not achieve a completely implicit approach to motor learning.

The retention of performance of the IO group under multitasking conditions can be related to the audio/visual presentation of the hand instrumentation technique. The partially independent nature of the visual and auditory working memories, resulting in less load on working memory, may have led to maintenance of performance.

The study findings do not support the original hypothesis and indicate that the method adopted for learning by observation with guidance was not unvarying with implicit learning approaches. The observational learning strategies used in this study seem to have overloaded working memory in terms of the amount and complexity of procedural knowledge. This overload for novice participants is suggested to increase their attentional demands between the various components of the same task, thereby leading to impairment of their performance.
